# Testing for hereditary thrombophilia: a retrospective analysis of testing referred to a national laboratory

**DOI:** 10.1186/1472-6890-8-3

**Published:** 2008-04-02

**Authors:** Brian R Jackson, Kyland Holmes, Amit Phansalkar, George M Rodgers

**Affiliations:** 1 University of Utah Department of Pathology, Salt Lake City, UT, USA; 2 ARUP Laboratories, Salt Lake City, UT, USA; 3 ARUP Institute for Clinical and Experimental Pathology, Salt Lake City, UT, USA

## Abstract

**Background:**

Predisposition to venous thrombosis may be assessed through testing for defects and/or deficiencies of a number of hereditary factors. There is potential for confusion about which of these tests are appropriate in which settings. At least one set of recommendations has been published to guide such testing, but it is unclear how widely these have been disseminated.

**Methods:**

We performed a retrospective analysis of laboratory orders and results at a national referral laboratory to gain insight into physicians' ordering practices, specifically comparing them against the ordering practices recommended by a 2002 College of American Pathologists (CAP) consensus conference on thrombophilia testing. Measurements included absolute and relative ordering volumes and positivity rates from approximately 200,000 thrombophilia tests performed from September 2005 through August 2006 at a national reference laboratory. Quality control data were used to estimate the proportion of samples that may have been affected by anticoagulant therapy. A sample of ordering laboratories was surveyed in order to assess potential measurement bias.

**Results:**

Total antigen assays for protein C, protein S and antithrombin were ordered almost as frequently as functional assays for these analytes. The DNA test for factor V Leiden was ordered much more often than the corresponding functional assay. In addition, relative positivity rates coupled with elevations in prothrombin time (PT) in many of these patients suggest that these tests are often ordered in the setting of oral anticoagulant therapy.

**Conclusion:**

In this real-world setting, testing for inherited thrombophilia is frequently at odds with the recommendations of the CAP consensus conference. There is a need for wider dissemination of concise thrombophilia testing guidelines.

## Background

Venous thrombosis is a common clinical problem. A national hospital discharge database estimates that in 2003, there were 177,000 discharges from U.S. hospitals with an ICD9-CM diagnosis code for pulmonary embolism (415.1) and 424,000 with a diagnosis code for venous thrombosis (453.0 through 453.9)[1]. A number of heritable disorders predisposing to thrombosis have been identified, including deficiencies and/or defects of protein C, protein S, antithrombin, factor V and factor II (prothrombin); patients presenting with deep vein thrombosis or pulmonary embolism are often tested for these defects. Given the range of tests, and particularly the existence of multiple tests for most of these analytes (e.g. protein S total antigen, free antigen and activity), there is potential for confusion about which tests to order and when to order them. Indeed, Somma et al. found that a significant proportion of orders appeared not to be clinically indicated in their study of 200 consecutive thrombophilia panel orders at an academic medical center[2].

The College of American Pathologists convened a consensus conference in 2002 to address diagnostic testing for thrombophilia, with findings published in series of articles in a pathology journal[3] as well as on the CAP website[4]. The findings are not currently published in the National Guidelines Clearinghouse[5]. For purposes of this study, the CAP recommendations were considered to represent diagnostic best practices as described by coagulation experts.

Among the large number of recommendations issued were several which address the choice of methodology for initial testing (Table 1). If physicians are ordering in a manner consistent with these recommendations, this should be reflected in the order volumes seen in the clinical laboratory. For example, the conference recommended that testing for antithrombin deficiency should begin with a functional assay[6]. If the patient is found to be deficient, then a total antigen assay may be useful in subclassifying the patient's defect. If this recommendation were followed, then the laboratory would expect the number of antithrombin total antigen assays ordered to be less than or equal to the number of positive (i.e. results less than the lower limit of the reference interval) antithrombin functional assays. Furthermore, the laboratory would not expect the functional and total antigen assays to be ordered on the same specimen in most cases.

**Table 1:** Selected recommendations of CAP consensus conference XXXVI: Diagnostic Issues in Thrombophilia

Initial testing for factor V Leiden may in most cases be appropriately performed by either direct DNA or functional (i.e. 2^nd ^generation activated protein C resistance (APC resistance) assays [[9,10]]
Initial testing for antithrombin deficiency should be via the functional assay, rather than the total antigen assay [6]
Initial testing for protein C deficiency should be via the functional assay, rather than the total antigen assay [7]
Initial testing for protein S deficiency should be via either the functional assay or the free antigen assay, rather than the total antigen assay [8]
It is preferable that testing for antithrombin, protein C or protein S deficiency take place after the patient has recovered from the acute thrombosis. [[6-8]]
It is preferable that testing for antithrombin, protein C or protein S deficiency take place when the patient is not on oral anticoagulant therapy [[6-8]].

Conference recommendations also addressed the timing of testing. Since anticoagulation and acute thrombosis can lower levels of protein C, protein S and antithrombin, it is preferable that testing for these be performed when the patient has recovered from the acute event and is not on anticoagulant therapy [6-8]. Anecdotal experience has suggested this recommendation is often not followed.

The purpose of our study was to estimate the extent to which thrombophilia test ordering patterns as observed at a national reference laboratory are consistent with diagnostic best practices as represented by the CAP consensus conference recommendations.

## Methods

We obtained the results of all protein C, protein S, antithrombin, factor V Leiden (FVL)/activated protein C (APC) resistance, and prothrombin G20210A assays performed at Associated Regional and University Pathologists (ARUP Laboratories) between September 1, 2005 and August 31, 2006. There were a total of 197,771 orders for these tests during this period. ARUP is a reference laboratory owned by the University of Utah that serves several hundred hospital and regional laboratory clients nationwide. ARUP maintains a long term data repository containing laboratory orders and results along with limited demographic data such as age and sex of the patient, but no clinical data.

To address the issue of overall test selection, we examined the relative ordering volumes for the different tests available for each disorder. For protein C and antithrombin, we calculated the ratio of activity tests to total antigen tests. The former were considered by the consensus conference to be appropriate as part of the initial workup of patients, whereas the latter were considered to be only appropriate in the followup of patients with a demonstrated deficiency[6,7]. For protein S, we calculated the ratio of activity tests plus free antigen tests to total antigen tests, since the conference considered protein S free antigen to be an alternative first-line test[8]. For activated protein C resistance, we calculated the ratio of APC resistance functional assays to FVL DNA-based assays; both the CAP conference recommendations[9] and an American College of Medical Genetics guideline[10] consider both assays to be first-line tests.

To address the issue of timing, we considered three different pieces of evidence: deficiency rates for different defects, concomitant deficiency of multiple vitamin K-dependent factors, and prothrombin time (PT) elevations.

Under the assumption that the patient population for each of these tests is similar, then the relative positivity rates should reflect the proportions of patients with thrombosis who have been found in other studies to have each of the studied defects. Most population prevalence studies have found V Leiden mutation to be roughly an order of magnitude more prevalent than protein S, protein C or antithrombin deficiencies[11] though it could be argued that studies based on healthy populations could over-represent the ratio of V Leiden mutation to these other defects in symptomatic patients because of the possibility of acquired deficiencies. At least one study, however, has confirmed significantly higher rates of V Leiden defects than protein C, protein S or antithrombin deficiency in patients with venous thrombosis[12]. If the ratio of V Leiden positivity in our data set were not substantially higher than protein C and protein S positivity, this could be suggestive of suboptimally timed specimens, e.g. specimens drawn during acute thrombotic episodes and/or during anticoagulant therapy. Since hereditary defects for these analytes are individually uncommon even in patients with thrombosis, results suggesting abnormalities of multiple analytes on the same specimen would likewise be evidence for suboptimal timing.

To further estimate what fraction of patients may have been on anticoagulant therapy, we analyzed prothrombin time (PT) and partial thromboplastin time results on plasma specimens for which were positive for protein C and/or protein S deficiency. (Note that for quality control purposes, ARUP performs a PT and PTT on every plasma specimen submitted for coagulation studies.) We set our threshold for defining PT elevation at a level equivalent to an international normalized ratio (INR) of 1.3. This cutoff was chosen to include the vast majority of patients on oral anticoagulant therapy while excluding most others. The cutoff likely missed some patients on early anticoagulant therapy, inadequate doses, and recently discontinued therapy who could still have had altered protein C and/or protein S results despite an INR less than 1.3.

Because ARUP is a primarily a referral laboratory, there were several potential sources of bias that could have affected the order volume ratios. One is that ARUP cannot definitively identify which tests were ordered as part of routine clinical care and which ones were ordered for other reasons, such as research studies or internal laboratory quality control. Evidence for such non-routine-care orders can be seen in the form of occasional transient increases in monthly order volumes from particular client laboratories. To qualitatively assess for the presence of such a bias we created a filtered data set as described below and compared test volume ratios derived from the raw data with the same ratios derived from the filtered data. Similar results in the two data sets would be evidence against a significant bias.

Under the assumption that a client's patient mix remained similar over the 12 month period, ARUP would expect to receive relatively constant proportions of orders for various tests from the client from month to month. The distribution of 10 different thrombophilia tests ordered by a client in a month was represented by a multinomial distribution ~(p1, p2, ... p10), with the numbers p1, ..., p10 representing the proportion of total volume for a particular client in a given month that was due to each of the 10 tests examined. For each client we compared these proportions from each month with those from every other month using the Multinomial Likelihood Ratio test under the null hypothesis that the proportions from any two months were equal. We then used the Holm test to adjust p-values for each client-month, discarding all client-months with a p value < 0.05 for any further inference;[13] 1.5% of all client-months were discarded at this step.

Another potential source of bias was that some of ARUP's client laboratories perform some of these tests in-house while referring the other tests to ARUP. Although ARUP does not routinely maintain data on its clients' test menus, CAP proficiency testing participation data[14] suggest that more laboratories perform functional assays for protein S, protein C and antithrombin than perform total antigen assays for the same proteins. (Note that the CAP Surveys program is one of several external proficiency testing programs that laboratories may enroll in to satisfy regulatory requirements.) To evaluate this bias, we surveyed a subset of clients to determine whether they were performing any of these tests in-house. The twenty clients with the highest volumes of orders for these tests as well as a random sample of all other clients comprising the top eighty percent of ARUP's order volume for these tests were queried either by fax, phone, or e-mail. Out of 46 clients contacted, 33 clients completed the survey. These 33 laboratories represented 19.3% of the total volume of approximately 200,000 tests analyzed in this study. The results of the survey are displayed in Table 2. For each analyte, the test order volume ratio was separately calculated for this set of 33 laboratories before and after excluding laboratories offering any in-house testing for that analyte.

**Table 2:** Results of client survey regarding in-house testing for thrombophilia tests of interest to this study.

Test	Percent of survey respondents performing tests in-house (%)
Protein C Functional	12.5
Protein C Total Ag	0.0
Protein S Functional	9.4
Protein S Free Ag	3.1
Protein S Total Ag	0.0
Antithrombin Functional	25.0
Antithrombin Total Ag	3.1
APC Resistance	12.5
Factor V Leiden by PCR	21.8

This study was performed on fully de-identified data using a protocol deemed by the University of Utah Institutional Review Board to be exempt from federal regulation.

## Results

Ratios of test order volumes are shown in Table 3. For protein C, protein S and antithrombin, second-line tests were ordered almost as often as first-line tests. The ratio for antithrombin was the most favorable of these, but total antigen assays still made up approximately one third of all antithrombin orders. For V Leiden/APC resistance, the functional assay was only ordered about one tenth as often as the DNA assay. The ratios were not substantially altered by filtering out orders from anomalous client-months. The ratios based on the 33 surveyed clients were similar to those based on the full data set, and were only modestly affected by filtering out data for clients performing testing in-house.

**Table 3:** Ratios of test order volumes by defect category (See Methods for explanation)

	Raw data (all clients)	Filtered data	All surveyed clients	Surveyed clients not testing in-house
Protein C Functional/Total Ag ratio	1.10	1.09	1.12	1.65
Protein S (Functional+Free Ag)/Total Ag ratio	1.28	1.27	1.21	2.10
Antithrombin Functional/Total Ag ratio	2.10	2.10	2.91	3.50
APC resistance/V Leiden DNA ratio	0.13	0.12	0.15	0.15

Positivity rates are shown in Table 4, along with mean age and sex distribution corresponding to each test. Of specimens on which tests for both protein C and protein S tests were ordered, 6.5% were positive (i.e. result below the reference interval on at least one assay) for both protein C and protein S deficiency. For specimens on which protein C, protein S and antithrombin tests were all ordered, 15.7% of these specimens had positive results for two of the three analytes; 1.2% had positive results for all three. From 21–54% of results indicating protein S or protein C deficiency were associated with an INR greater than 1.3 (Table 4).

**Table 4:** Positivity rates and patient characteristics by test ordered.*

	Positivity	Fraction of positives which also had INR>1.3	Median Age	% Female
Protein S Functional	17.7%	21.2%	46	65.7%
Protein S Total Ag	4.8%	40.5%	47	63.0%
Protein S Free Ag	18.5%	25.0%	39	67.4%
Protein C Functional	13.7%	53.8%	46	65.4%
Protein C Total Ag	12.9%	33.3%	47	62.9%
Antithrombin Functional	7.5%	N/A	46	65.8%
Antithrombin Antigen	14.3%	N/A	46	65.3%
APC resistance	17.7%	N/A	46	65.7%
V Leiden mutation	12.3%	N/A	47	64.1%
Prothrombin G20210A mutation	4.9%	N/A	46	64.1%

*Positivity for V Leiden and prothrombin mutations includes both heterozygotes and homozygotes; for the remaining assays it includes all results falling below the lower limit of the reported reference interval.

## Discussion

The ordering patterns for thrombophilia tests observed at our laboratory during the study period do not appear to be consistent with practices recommended by the CAP consensus conference. According to these recommendations, total antigen tests have a much more limited role than the other assays for protein S, protein C and antithrombin. The fact that they are ordered within an order of magnitude as often as first-line tests is evidence that clinicians are not aware of the appropriate role of these tests or of the CAP consensus conference recommendations. Likewise, the fact that the DNA test for FVL is ordered almost ten times as often as the functional APC resistance assay (which has a lower cost and faster turnaround time) suggests that clinicians are either unaware of the functional assay or else unaware that it is considered an equally appropriate first-line test in most cases.

Positivity rates for the antithrombin, protein C and protein S assays were much higher than would be expected relative to the observed prevalence of FVL and prothrombin G20210A mutations, as well as previously published data on the relative frequencies of inherited thrombophilia defects. Because the V Leiden and prothrombin G20210A mutations are both DNA-based tests, results of these tests directly measure the heritable defect frequencies in the population being tested. Assuming that the patient population for all these assays is reasonably homogeneous, the expected frequencies for inherited deficiencies of protein C, protein S and antithrombin would be roughly an order of magnitude lower. The high positivity rates for these latter assays, combined with the high rates of concomitant positivity for multiple analytes as well as the high frequency of elevated INR on these specimens (Table 4), suggest that many of these positive results are actually false positives due to suboptimally timed testing.

Prior studies have shown variability in the use of these tests. Bushnell et al. identified non-evidence-based ordering by neurologists on patients with ischemic stroke, based on both observation[15] and survey[16]. Robertorye calculated test order ratios at ARUP Laboratories in 1998 and 1999 for protein C, protein S, antithrombin and FVL, with similar results to those reported in this paper[17]. In this previous study, which was based on raw order volumes only, the functional assay to antigen assay ratios were 0.53, 0.54 and 2.59 for protein C, protein S and antithrombin, respectively. The analogous ratio for FVL was 3.71. Because of fluctuations in ARUP's client mix it is difficult to draw conclusions from the changes in these numbers over time; nonetheless, both sets of results demonstrate a considerable degree of questionable ordering.

The appropriate role of thrombophilia testing as a whole has also been discussed in the literature. The CAP conference recommended that based on available evidence, anticoagulant therapy for thrombosis should be based primarily on clinical factors rather than laboratory identification of one of the above defects. Others have issued similar recommendations[18]. Proposed indications for testing include family counseling (in the case of heritable defects) and clarification of etiology. Most authors advocate selective testing in young patients, those with a relevant family history and/or those with unusual or severe presentations, though specific recommendations differ by author[19-23]. It may be the case, in fact, that the most widespread problem in thrombophilia testing is not test selection per se, but rather ordering *any *of these tests in the first place on patients who have clear nonhereditary precipitants of thrombosis[2]. Given our data set, we could not determine the proportion of patients with thrombosis who were worked up for hereditary thrombophilia, nor could we determine the extent to which test results were used to drive therapy.

The financial implications of inappropriate thrombophilia testing are considerable. At ARUP we have observed that thrombophilia tests account for a disproportionately high fraction of our clients' sendout test budgets. Ordering antigenic and functional tests for protein C, protein S and antithrombin, along with a DNA test for the V Leiden mutation on a hypothetical Medicare patient in Utah would cost Medicare $141.85 in reimbursement based on 2005 rates[24]. Billed charges would be considerably higher. At least as important as direct test costs are the downstream costs associated with follow up care. A false-positive protein S result, for example, might lead to repeat diagnostic testing, additional office visits and/or anticoagulant therapy. The sum of these costs could dwarf the cost of the initial protein S assay.

The implications for patient safety are arguably more important than the financial implications. To the extent that either anticoagulant therapy or genetic counseling were to rely on incomplete or faulty laboratory diagnosis, a patient may be put at risk.

The fact that some of ARUP's clients perform the functional tests in-house indicates a likely bias in our full data set. We believe this bias to be modest, however. First, basing the volume ratios only on clients known to not perform any of the relevant tests in-house did not markedly alter our findings (Table 3). Second, although CAP Survey participation data indicates that laboratories more often perform functional than total antigen testing for these analytes, it also indicates that only a minority of coagulation laboratories perform any of the tests in this study at all.

Another limitation of this study design was the lack of linked clinical data. In this absence we could not separate clinicians' ordering decisions from the interpretation of these orders at the local laboratory. For example, if a clinician were to write "Protein C" on a requisition form, and if this were interpreted by local laboratory personnel as an order for the total antigen test, then the latter order would be the only one identified in this study. Also, we assumed that these tests were ordered for venous thromboses, but it is possible that many of these tests could have been ordered for arterial thromboses and/or other diagnoses[16]. Finally, we could not directly assess which patient may have had an acute thrombosis or been on anticoagulant therapy at the time of testing; we could only infer this indirectly through prothrombin time results and relative positivity rates.

Out of 179 recommendations from the CAP consensus conference[3], our study only considered those which concerned test ordering and for which compliance could be assessed through analysis of laboratory data alone, i.e. in the absence of clinical correlation. The findings reported here therefore do not apply to the entire set of conference recommendations. For example, many of the recommendations related to testing methodology were directed toward laboratory professionals, and these may have been more widely adopted. It seems reasonable to assert, however, that our findings reflect a general lack of adoption of conference recommendations by ordering clinicians, possibly due to a lack of awareness.

Recommendations such as those generated by this CAP consensus conference fill a critical role in aggregating and synthesizing medical knowledge[25]. It is true that the literature on adoption of clinical practice guidelines into routine practice shows a mixed record[18]; nonetheless, a reasonable step toward broader dissemination of these thrombophilia test ordering recommendations might be consolidation into a single concise guideline for inclusion in resources such as the National Guideline Clearinghouse[5]. As a complementary approach, laboratories might consider incorporating some of this guidance into interpretive statements attached as footnotes to individual laboratory results. Such statements might include clarifying that the reference intervals are valid only for individual free of thrombosis and not on anticoagulant therapy.

## Conclusion

Our findings suggest that a substantial proportion of thrombophilia ordering is not consistent with the recommendations of the CAP consensus conference. Some patients may receive suboptimal care as a result. Laboratories and health care provider organizations need better mechanisms to promote appropriate utilization of thrombophilia tests.

## Competing interests

Brian Jackson and George Rodgers are consultants for ARUP Laboratories.

Kyland Holmes and Amit Phansalkar were employees of ARUP Laboratories at the time of manuscript preparation.

## Authors' contributions

BRJ conceived the study, analyzed data, and drafted the manuscript. KH conducted the laboratory surveys and assisted in manuscript revisions. AP analyzed data, designed statistical tests and contributed to drafting the manuscript. GR participated in study design, interpretation of results and drafting of manuscript. All authors read and approved the final manuscript.

## Pre-publication history

The pre-publication history for this paper can be accessed here:


